# A Case Illustrative of the Effects of the Colchicum Autumnale

**Published:** 1830

**Authors:** R. J. Buckner

**Affiliations:** Brown Co. Ohio


Art. III.—*A case illustrative of the Effects of the Colchicum Autumnale.*—By Dr. R. J. Buckner, of Brown Co. Ohio.

During the summer of 1828, in the vicinity of Georgetown, Brown county, Ohio, those who resided near the watercourses, in the neighborhood of mill ponds, or on low marshy grounds, were very generally attacked with remittent bilious fever, which, with a few exceptions, was of a mild character, yielding readily to the usual mode of treatment.

Towards the close of the month of August, it became dry, and warm, about which time, fluxes and dysenteric fever, became very prevalent, assumed rather an inflammatory character, and when not met, at an early stage, with bold and energetic remedies, became protracted; with something of a typhoid type. In the month of September, the weather was very changeable with frequent showers of rain and cold nights; about which time appeared a few cases of acute Rheumatism highly inflammatory, to one of which I was called in the person of Mr. E. Thompson.

The patient was near the age of fifty; inured to hard labor about a mill, and had a few days previously, been engaged in repairing his mill dam, and plunged frequently in the water when very warm, and perspiring freel y:—to which causes I attributed his disease. He was a man of temperate habits, in regard to spiritous liquor, but indulged freely in animal diet; his temperament rather bilious.

When I first saw him, he was evidently laboring under all the symptoms of an ardent fever; skin hot and parched—face highly flushed—pain in the head and back—pulse full hard and burning—thirst intolerable—tongue foul, inclining to a brown appearance in the centre, edges very red: he complained of much pain in the lower extremities, particularly the right knee and ankle, which joints were considerably swollen—his right arm was also considerably affected. On the slightest touch, the pain was excruciating and seemed confined to the faciæ of the muscles and the tendinons envelopes of the joints. I considered his case a well marked, acute Rheumatism. I had him raised up in bed and extracted 20 ℥ of blood, which produced slight nausea and a tendency to syncope. The blood exhibited strong marks of inflamation. I then administered a free cathartic of calomel and jalap, which was [illegible]owed after an interval of about six hours, by sulph. magn[illegible]s. ℥jj, tart. antimo. grs. iv, dissolved in half a pint of warm water—a wine glass full was taken every hour, which produced copious alvine evacuations of very dark, bilious matter.

In the course of fifteen or eighteen hours, I visited him. Very little abatement of the pain, except of the head and back, which was removed. Pulse still full and rather hard—skin hot and dry;—bled him again to 16℥ sitting erect in bed—pulse became soft—slight moisture appeared on the skin. I now applied blisters to his wrists and knees, and left Dover's powder to be taken every three hours, in a little warm tea, to promote diaphoresis.

Next morning found him much better. Pain not so excruciating—perspired,freely during the latter part of the night—for several days but little alteration in the symptoms. The disease them assuming the sub-acute form, I ordered the blisters to be reapplied to the parts—Dover's powder continued—very little fever now, except at night, when the evening paroxysms would return, regularly and severely, which prevented his sleeping: indeed he enjoyed no sleep, except when he could be prevailed on to take the Dover's powder, in sufficiently large doses to produce diaphoresis, which always mitigated the pain and procured him some repose; but from the nausea they produced, his protracted sufferings, &c. he became extremely averse to taking any more medicine. I had urged upon him, for several days previously, the absolute necessity of taking mercury in some form, with a view to mercurialize the system, but this he utterly refused, being unhappily under the influence of the most violent prejudices against the use of mercury in any form whatever. The disease absolutely resisted the mode of treatment which had hitherto been pursued, and continued its march unrestrained, harassing him with lancinating pains and sleepless nights, until he became very much reduced, when it began to exhibit considerable tendency to metastasis—the stomach, diaphragm, intestines, and respiratory muscles, all showing, successively, strong symptoms of the disease, I became alarmed for the result. I requested him to send for a consulting physician, in hopes a consultation might induce him to take medicine, and submit to what I supposed to be the proper mode of treatment. Having at length consented to this proposal, an aged and respectable gentleman of the medical profession was consulted, who advised by all means to administer calomel opium, and squills, so as to mercurialize the system without delay, and to continue the blisters, diaphoretics, &c. to all which the patient obstinately objected. Such was the strength of his preconceived objections against all mercurial preparations: but he observed—to use his own words—"If I can get some kind of drops to ease these pains, I will try them." It then occured to me to try the tinct. colchici. I

suggested it to the consulting physician, who replied, that under all circumstances, he would recommend its trial. I had some of the tincture prepared according to the following formula—℞. "Pulv. colchici ℥ss, Alcohol ℥iv, let it stand for 10 days then filter through paper." Of this tinct. I ordered about a drachm three times a day. The second potion produced slight vertigo, and burning heat of the fauces and stomach, followed by a pretty free discharge of *Saliva*. His pain was, for a short time, rather increased;—as he expressed it, "I am pained all over." The third or fourth dose produced complete *Ptyalism*—a gentle moisture of the skin, and perfect relief from pain.

The following day I visited him again, to encourage him to persevere in taking the medicine, intending if permitted, to give it a fair trial, and found him as just stated—skin soft—free from pain and spitting copiously—the secretions generally established, particularly those of the kidneys—skin, and as I have stated, salivary glands. As soon as I entered his room, he said to me—"You have deceived me at last—you have *salivated* me." I assured him I had not given him any medicine that was calculated to produce that effect:—he replied, "you need not deny it—I don't care—I am evidently better—I could have slept all night, if I had not been disturbed by the spitting—I think I must have spit half a gallon since last evening." I was very much surprised at this account as well as what I witnessed myself, for in ten years of pretty extensive practice, but few cases of ptyalism from the exhibition of mercury, have come under my observation, in which the saliva flowed more freely.

It will here be recollected, that for several days prior to the use of the colchicum, she had used nothing but Dover's powder, and an occasional purgative of neutral salts or oil; that he had taken but a single dose of calomel, and that combined with jalap, followed and briskly worked off by salts; so that I am led to attribute the ptyalism solely to the *Colchicum*. Besides the mercurial fœtor of the breath was not at all perceivable. I continued the colchicum for eight or ten days, during which time, the patient convalesced rapidly—the pain having left him in twenty-four hours after he commenced using it—the swelling of the joints gradually disappeared, leaving him perfectly free from every symptom of the disease.

His debility and emaciation being very great, I now prescribed as tonics, the sulph. quinæ, wine, and a generous diet, —under this course, he soon regained his usual health and strength, and has not since experienced any returning symptoms of the disease.

Pleased with the effects of the Colchicum in this case, I have since tried it in a number of diseases—particularly Rheumatism, Sciatiea, Dropsy, Paralysis, &c. and in many of these cases, with manifest good effect; but in none was its action so immediately and completely successful, as in that of Thompson: and it is due to candour to observe, that, in a few instances, I have given it, without discovering that it produced any effect whatever. Whether, in those cases where it failed, it was owing to the disease not being of the class which it is best calculated to relieve, or whether the stage of the disease—time and other circumstances not being propitious to its operation—it failed of success, I leave to be decided by future observation.

I have given the above detailed report of the character of the disease of Thompson—the stage and circumstances, (comprising the previous treatment,) under which the Colchicum was administered, and I think we may safely conclude that in that particular case, it was the efficient remedial agent.

I have no where seen its peculiar action on the salivary glands, stated as one of its effects. But in my own trials, where it was decidedly beneficial, it has invariably produced the following effects:—An increased flow of saliva, in a greater or less degree, reduced action of the heart and arteries; and a decided mitigation of pain.

From this little discovery, and others of a more important character, in the science of medicine, which are daily making, I am led to concur with the illustrious Dr. Armstrong, in a prediction, which I cannot forbear quoting in his own language. "I have no doubt," says he, "that some great discovery will be made in Therapeuties, by which the treatment of most if not all febrile diseases will be made much more simple and successful; for we frequently now employ only bleeding, purging and other ordinary means—not because they are the best which can be *discovered*, but merely because they are the best which we *know*, in the present state of our imperfect knowledge.

"From the great power which narcoties possess over the nervous and thence over the vascular system, it is highly probable, that some agents of *that* tribe may yet be found by which the dominion of medicine may be much extended. Whether *Colchicum* be one of these, the experience of others, hereafter, must determine; but I have seen enough of its effects, to be convinced that few articles of materia medica are more deserving of consideration."

The medical accounts of this drug, are so exceedingly defective, that a great deal remains to be known; not as to its practical operation, only; but as to the best season for gathering, and the best mode of preparing it for use.

If the few desultory remarks offered in this humble paper, should be the means of calling the attention of the Faculty, to the further investigation of this subject, and if that investigation shall happily result in establishing the salutary efficacy of this powerful agent in the control of one or more, of the many diseases which afflict the human family, my fondest, and most sanguine expectations will be realized.

December, 1829.

